# A cross-dataset harmonized intrusion detection framework with statistically validated multi-model learning

**DOI:** 10.1371/journal.pone.0346982

**Published:** 2026-04-20

**Authors:** Shailendra Mishra, Naif S. Alshammari, Hashim Hussain, Ruba Ahmed Alfahidah

**Affiliations:** 1 Department of Computer Engineering, College of Computer and Information Sciences, Majmaah University, Al Majmaah, Saudi Arabia; 2 Department of Computer Science, College of Computer and Information Sciences, Majmaah University, Al Majmaah, Saudi Arabia; 3 Department of Information Technology, College of Computer and Information Sciences, Majmaah University, Al Majmaah, Saudi Arabia; Oakland University, UNITED STATES OF AMERICA

## Abstract

Intrusion Detection Systems (IDS) are considered critical security tools in ensuring network infrastructure security. However, recent studies on machine learning-based IDS systems are often constrained by their heavy dependence on a single dataset, lack of reproducibility, and lack of transparency in evaluating their performance. In addressing these challenges, a unified and transparent framework for evaluating IDS systems is proposed, which focuses on integrating feature harmonization, multi-model benchmarking, and statistical validation. In achieving this objective, a preprocessing pipeline is designed to harmonize features of both legacy and contemporary network intrusion datasets, i.e., NSL-KDD and CICIDS2017, respectively. This framework will assess various learning models, including supervised, unsupervised, deep learning, and ensemble-based models, through cross-validation and statistical tests such as Wilcoxon signed-rank, McNemar’s, and DeLong tests. Experimental results demonstrate that the Random Forest model performs best in terms of performance metrics, i.e., 98.0% accuracy and 97.0% F1-score on the harmonized data set. Moreover, feature harmonization is found to be the most important factor in improving performance using ablation analysis. Besides, a novel approach of using a cryptographic logging mechanism using SHA-256 hash chaining is proposed for tamper-evident traceability and reproducibility of results in experiments, though it is not as effective as using a blockchain-based approach. Although effective in its application, it is based on manual feature alignment and hence might not be effective in highly heterogeneous data sets.This work provides a unified, reproducible, and statistically grounded framework for evaluating IDS systems, focusing on generalization and transparency in cybersecurity research.

## Introduction

Due to the rapid growth of cyber threats, it has become a necessity to have intrusion detection systems (IDS) as a vital part of the security infrastructure. The global cost of cybercrimes is estimated to reach trillions of dollars annually [1].The traditional approach to intrusion detection systems, which relies on signature-based detection systems, can only efficiently identify known threats but lacks the capability to identify unknown threats. This led to the use of machine learning and AI technologies to identify unknown threats using complex patterns [2–5].The recent advancements in intrusion detection systems have been in AI-based zero trust architecture for industrial IoT environments. This reflects a new direction in security systems that are adaptive in nature [6].

While substantial progress has been made in machine learning-based intrusion detection systems, some of the underlying issues still remain unresolved. The first issue is that existing intrusion detection systems heavily rely on a set of predefined benchmark datasets, which results in models that cannot generalize well in different environments. Moreover, some of the underlying models lack uniformity in data preprocessing techniques, evaluation protocols, and statistical validation. Additionally, most of the existing models lack transparency in tracking experiments, making it hard to reproduce results.

On the other hand, more research is being conducted on deep learning models and optimization techniques. Although deep learning models have shown high accuracy in intrusion detection systems, most of the models lack rigorous evaluation and comparison of results with traditional and ensemble-based models. Moreover, little research has been conducted on generalizing models on different datasets, which is important in real-world intrusion detection systems in dynamic and evolving environments [7–10].

To overcome these challenges, in this research, a transparent and extensible framework for evaluation of IDS systems with three main goals is proposed:

1. Cross-dataset feature harmonization to allow for the consistent representation of features across different legacy and contemporary datasets;
2. Unified multi-model benchmarking to allow for the evaluation of different types of models, including supervised, unsupervised, deep learning, and ensemble models;
3. Reproducible and verifiable experimentation through the use of structured tracking and tamper-evident logging.

The main focus of this paper is to design a unified framework for experimental evaluation instead of designing a novel learning algorithm. The paper only considers offline evaluation using two popular benchmarking datasets: NSL-KDD and CICIDS2017, which correspond to traditional and recent network traffic. It seeks to enhance dataset generalization, fairness in comparison to various learning paradigms, and reproducibility using standardized preprocessing, statistical verification, and logging mechanisms. However, real-time deployment and adaptation are out of the paper’s scope.

The proposed framework combines NSL-KDD [11] and CICIDS2017 [12] by utilizing semantic feature alignment and normalization. It supports joint training and transfer evaluation of different learning paradigms via repeated cross-validation and cross-dataset testing. Statistically significant evaluation is performed by utilizing the Wilcoxon Signed-Rank, McNemar’s, and DeLong tests [13]. Moreover, a lightweight cryptographic hash-chained logging approach is utilized for reproducibility and auditability while mitigating the overheads associated with blockchain-based techniques [14–16].

The novelty of this research is in the development of a unified framework that incorporates several aspects of intrusion detection system (IDS) evaluation that have not been sufficiently explored and are critical for a more effective IDS. These aspects are: (i) a semantic feature harmonization approach for cross-dataset learning on heterogeneous IDS datasets, addressing dataset dependency and generalization issues; (ii) a unified multi-paradigm IDS benchmarking framework for evaluating various IDS paradigms, such as supervised, unsupervised, deep learning, and ensemble methods, in a unified framework and preprocessing and evaluation pipeline; (iii) a statistically sound validation approach that ensures the efficacy of the proposed improvements using Wilcoxon Signed-Rank, McNemar’s, and DeLong tests; and (iv) a lightweight and efficient approach to a tamper-evident and secure logging mechanism that leverages a hash chain of SHA-256 hashes for IDS evaluation. This research is distinct from previous research in that it presents a comprehensive and transparent framework for IDS research, addressing aspects such as IDS evaluation, generalization, fairness, and reproducibility.

The remainder of this study is organized as follows: Section 2 reviews related literature, Section 3 describes the proposed methodology in detail, Section 4 presents the experimental results and analysis, Section 5 discusses implications and limitations, and Section 6 concludes the work with directions for future research.

## Literature review

### Machine learning in intrusion detection systems

The rise in the complexity of cyber-attacks, including DDoS attacks, Advanced Persistent Threats (APTs), and botnets, has fueled the use of machine learning (ML) techniques for the detection of new and complex cyber attacks in an automated manner [4,5]. Although the early benchmark datasets, such as KDD Cup ‘99 and its improved version NSL-KDD, were designed for the implementation of supervised learning techniques such as SVM and Random Forest algorithms, they were found to be ineffective in dealing with the new diversified traffic patterns [5,17]. In contrast, the latest flow-based datasets, including CICIDS2017, BoT-IoT, and IoTID20, are designed to reflect the new realities of enterprise and IoT environments and allow the implementation of advanced techniques such as the use of a hybrid of clustering and classification techniques [18], statistical feature selection techniques [19], and deep learning hybrids [8,20–22].

### Persistent challenges

In spite of these advancements, some of the limitations persist in ML-based IDS research, which are as follows:

- Cross-dataset generalization: The ML models are often trained on a dataset and fail to generalize on other network environments [21,22].
- Feature inconsistency: The lack of standardization in feature definition and preprocessing makes it difficult to compare and reproduce results across various studies [21,22].
- High-dimensional and imbalanced data: The performance of unsupervised and anomaly-based ML models is not satisfactory on large and highly imbalanced data sets containing various attack classes [17,21,23].
- Limited auditability: In ML-based IDS, there is a lack of incorporation of tamper-evident and secure auditing mechanisms for traceability of results and experiments [14].

### Recent research directions

Recent research works have addressed these issues. Using lightweight feature selection and genetic algorithms/correlation-based filtering, detection rates have surpassed 99% on IoT datasets [8,19]. Metaheuristics such as PSO, Jaya, and SSA have shown improvement in convergence and generalization capabilities of LSTM and CNN-LSTM architectures [9]. Ensemble and hybrid methods using kNN, SVM, RF, and deep autoencoders have shown robust performance on various datasets [24,25]. Quantum-inspired least square SVM and optimized DTL have shown very high accuracy rates, often 99% and beyond, on NSL KDD and CICIDS datasets [10,26].

Recent studies have been conducted to investigate the integration of blockchain technology with ML for improving the secure processing of data in Internet of Medical Things environments. For example, the application of blockchain-based architectures with SVM-based models was found to enhance the robustness and integrity of the secure processing of data in healthcare-based IDS environments [27].

In the same context, new authentication frameworks based on blockchain technology and integrated with edge computing were proposed for improving scalability, security, and real-time processing capabilities for Internet of Medical Things environments [28].

However, there are critical limitations that are evident in such studies. For instance, although Altulaihan et al. (2024) [8] attained 99.94–100% accuracy in the evaluation of the proposed DT/RF with GA and CFS on the IoTID20 dataset, the evaluation was conducted on a single dataset without cross-validation across different datasets. Moreover, although Dash et al. (2025) [9] attained 97.89–98.95% recall in the evaluation of the proposed optimized LSTM on NSL-KDD, CICIDS2017, and BoT-IoT datasets, the evaluation was conducted without rigorous statistical evaluation and auditability.

The emerging trends in AI-based intrusion detection systems focus on incorporating features such as transparency and trust into the system. These include privacy-preserving federated learning [29,30], explainable AI via SHAP and LIME [14,16,31], and lightweight cryptographic tamper-evident logging [14,15].No comprehensive unified framework yet exists that simultaneously integrates cross-dataset feature harmonization, multi-paradigm benchmarking (supervised, unsupervised, deep, ensemble), rigorous statistical validation, and tamper-evident experiment logging.

Emerging approaches combining generative artificial intelligence with intelligent monitoring systems further demonstrate the convergence of AI-driven technologies in healthcare and cyber-physical environments, enabling more adaptive and personalized security and decision-making systems [32]

These recent developments highlight the growing convergence of machine learning, blockchain, and AI-driven security mechanisms. However, the issues of cross-dataset generalization, evaluation standards, and experimental reproducibility are not sufficiently addressed, thus necessitating the development of a unified evaluation framework for IDS.

### Research gaps and positioning of the present work

The majority of the existing works are highly dependent on single dataset evaluation, repeat preprocessing of each dataset, and under-emphasize the role of unsupervised methods in high-dimensional and imbalanced scenarios [14,21,19,33,34]. Table 1 presents a comparative analysis of some of the latest works in the area of IDS, including the datasets, performance, innovations, and limitations of each work.

**Table 1:** Comparative analysis of selected IDS studies.

Citation	Dataset	Performance Metrics (Acc / Prec / Rec / F1%)	Contributions	Potential Constraints
**Vinayakumar et al. (2019) [2]**	NSL-KDD	~93% (DNN Acc); Overall >90%	Scalable hybrid deep learning IDS (scale-hybrid-IDS-AlertNet) for real-time attack detection	Older dataset; evolving malware not represented; manual feature engineering
**Maseer et al. (2021) [7]**	CICIDS2017	DT: 99.49%; kNN: 99.52%; CNN: 99.47%; RF: 99.30%; SVM: 75.21%	Broad ML/DL benchmarking on CICIDS2017	Metric inconsistency; limited feature engineering discussion
**Altulaihan et al. (2024) [8]**	IoTID20	DT: 99.94%; RF: 99.95%; SVM: 99.71%; kNN: 99.81% (No FS) DT/RF: 100% (GA); DT/RF: 99.99% (CFS)	ML-based IDS with GA and CFS feature selection for IoT DoS detection	Dataset specificity; redundancy in IoT features; lightweight deployment challenges
**Dash et al. (2025) [9]**	NSL-KDD; CICIDS2017; BoT-IoT	Acc 97.89; Rec 98.95 (SSA-LSTM)	LSTM optimized via PSO, JAYA, and SSA metaheuristics	Limited optimizer comparison; high training complexity
**Waghmode et al. (2025) [10]**	NSL-KDD; CICIDS2017	NSL-KDD: Acc 99.3%; CICIDS2017: Acc 99.5%	Optimized LS-SVM with feature selection; lightweight ML alternative to DL	Kernel sensitivity; hyperparameter tuning required
**Kumar et al. (2024) [14]**	ToN-IoT; IoT Healthcare	ToN-IoT: Acc 97.27, Prec 94.00, Rec 98.50, F1 96.20 Healthcare: Acc 89.62, Prec 99.13, Rec 76.18, F1 86.15	Blockchain (C-PoA) + PSLSTM + attention + SHAP explainability	Performance varies by domain; lower recall in healthcare scenario
**Meidan et al. (2020) [22]**	N-BaIoT	TPR ≈ 100%	Device-specific deep autoencoders for IoT botnet detection	Limited real-world diversity; missing full metric reporting; resource overhead concerns
**Aziz & Bestak (2024) [18]**	Call Detail Records (CDR)	Acc 96.1; Prec 0.421; Rec 0.727; F1 0.533	K-means clustering for telecom anomaly detection	Low precision; cluster labeling ambiguity; unsupervised instability
**Mhamdi & Isa (2024) [25]**	NSL-KDD (SDN)	Detection Rate 98%; FPR 1.85%	Hybrid Deep Autoencoder + Random Forest (DAERF) for SDN security	Computational overhead; SDN-specific evaluation
**Alsubaei et al. (2025) [33]**	IoT datasets	~99% Acc	Deep learning IDS for IoT with optimized architecture	Generalization across non-IoT traffic not tested
**Abu Al- Haija & Odeh (2023) [35]**	NSL-KDD	PCA/RP variants: 74–82%	Dimensionality reduction comparison (PCA, LDA, RP) for IDS	Lower performance; outdated dataset limitations
**Heidari et al. (2023) [36]**	IoT Drone Networks	Acc 98.7; Prec 97.9; Rec 98.2; F1 98.0	Blockchain + RBF neural network for secure drone IDS	Scalability and blockchain overhead in large deployments

Table 1, reveals a clear pattern: while individual-dataset accuracies are often impressive, cross-dataset generalization, standardized preprocessing, unsupervised stability, and experimental auditability are rarely addressed together. This work addresses these gaps by proposing a semantic feature harmonization pipeline to merge NSL-KDD and CICIDS2017, supporting unified multi-paradigm evaluation, feature selection based on mutual information, repeated cross-validation with statistical significance testing, and light-weight SHA-256 hash-chained tamper-evident logging for full reproducibility and traceability.

## Research methods

This section presents the methodological framework used for designing, executing, and assessing the proposed unified intrusion detection system (IDS). This framework attempts to resolve various issues associated with existing IDS-related literature, which were based on dataset dependency, preprocessing variability, lack of cross-paradigm evaluation, and transparency.

### Research objectives

The primary objective was to build binary models for intrusion detection using various paradigms of machine learning and evaluate them using a unified dataset derived from NSL-KDD and CICIDS2017 datasets. The secondary objective was to incorporate a simple cryptographic mechanism for traceable configuration and prediction without exaggerating the idea of blockchain technology [37].

### Framework overview

The framework integrates heterogeneous benchmark datasets, multiple learning paradigms, and reproducible evaluation via cryptographic logging. The workflow comprises six main stages:

1. Data acquisition and preprocessing
2. Cross-dataset feature harmonization
3. Feature selection and normalization
4. Model development across paradigms
5. Cross-validation and cross-dataset evaluation
6. Tamper-evident logging of metadata and predictions

**Fig 1:**
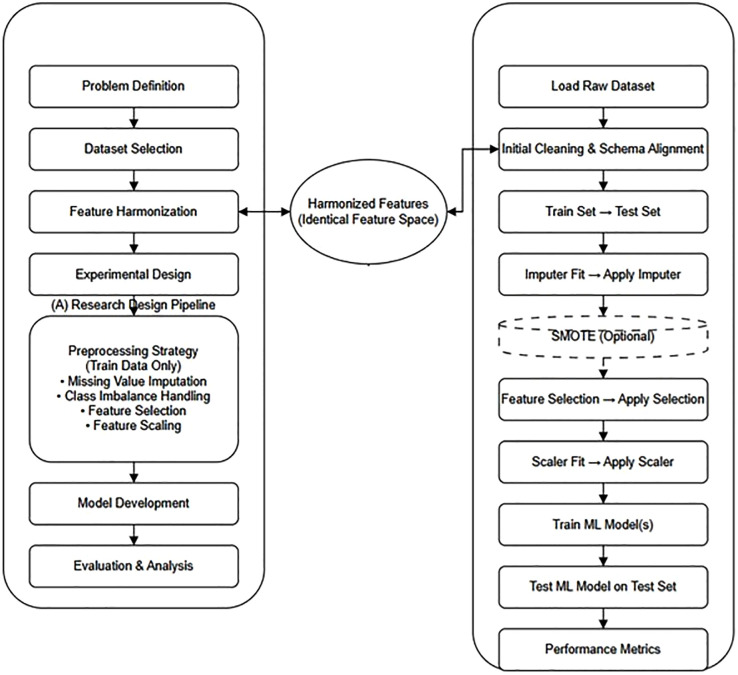
Proposed system architecture.

Fig 1 illustrates the system-level architecture. Both research design and implementation pipelines enforce a unified feature representation.

Two popular intrusion detection datasets were used. NSL-KDD is an improved variant of the KDD Cup ‘99 dataset, designed to reduce redundancy and class imbalance, which reduces data redundancy and imbalance. It contains connection-oriented features such as protocol type, service, flag, and calculated statistics. It is based on the KDD Cup ‘99 dataset but is improved to reduce data imbalance and redundancy. CICIDS2017 is a recent flow-based dataset that represents the characteristics of modern enterprise network traffic and various types of modern attacks, such as denial of service, brute force, infiltration, and web attacks. It contains time and statistical features derived from packet capture data. For the purpose of experimental evaluation, 100,000 instances were used, which were derived from the data.

### Data acquisition

Two widely used intrusion detection datasets were selected:

- NSL-KDD [11]: An improved variant of KDD Cup ‘99, reducing redundancy and imbalance. It contains connection-oriented features (e.g., protocol type, service, flag, derived statistics) and serves as a legacy benchmark for historical comparison.
- CICIDS2017 [12]: It is a recent flow-based data set that represents modern enterprise world attacks and normal traffic, such as DoS, brute force, infiltration, and web attacks. It also includes time and statistical features based on real-world packet captures, making it relevant to recent network environments.

The datasets were selected for this study based on their complementary characteristics. NSL-KDD is a standardized data set for representing traditional world attacks and normal traffic, while CICIDS2017 represents recent world attacks and normal traffic. These characteristics make it possible to perform robust cross-era generalization tests.

### Per-dataset class distribution (before merging)

The class distribution of each data set is presented before merging, shown in Table 2, in order to highlight the characteristics of class imbalance in each data set. It is evident that NSL-KDD is well-balanced after preprocessing, whereas CICIDS2017 is highly imbalanced in its class distribution, with more normal traffic instances.

**Table 2:** Class distribution before merging.

Dataset	Benign	Attack	Notes
NSL-KDD	~53,250	~46,750	Nearly balanced
CICIDS2017	~84,100	~15,900	Highly imbalanced

For computational purposes and to ensure a balanced representation in both merged experiments, 100,000 instances were sampled (50,000 from each dataset after stratification by class), given that NSL-KDD training data has ~ 126,000 records and CICIDS2017 has over 2.8 million flows [sources: UNB CIC Datasets documentation].

### Attack-category breakdown (before binarization)

Attack instances were categorized into different categories prior to binarization to ensure interpretability of the diversity of attacks (Table 3). NSL-KDD data has categories that include DoS, Probe, R2L, U2R, among others. CICIDS2017 data has more recent categories of attacks that include DoS/DDoS, brute force attacks, infiltration attacks, web attacks, etc.

**Table 3:** Attack category distribution (pre-binarization).

Dataset	Major Attack Categories
NSL-KDD	DoS, Probe, R2L, U2R
CICIDS2017	DoS/DDoS, Brute Force, Infiltration, Web Attacks

### Cross-dataset feature harmonization

The NSL-KDD and CICIDS2017 datasets differ significantly in their feature definition and granularity. A semantic alignment approach aligned semantically similar features based on their shared behavioral interpretation. For example, traffic volume features such as “src_bytes” (in NSL-KDD, cumulative) and “Flow Bytes/s” (in CICIDS2017, normalized to a rate) are aligned since they both describe patterns of intensity associated with volumetric attacks. To reduce distortions caused by structural differences, each dataset was normalized individually before fusion (Table 4).

**Table 4:** Cross-dataset feature harmonization.

Behavioral Concept	NSL-KDD Feature	CICIDS2017 Feature	Harmonization Strategy
**Traffic volume**	src_bytes, dst_bytes	Flow Bytes/s, Flow Packets/s	Converted to rate-independent z-scores within each dataset before merging
**Connection duration**	duration	Flow Duration	Log transformation + z-score normalization
**Packet count behavior**	count, srv_count	Total Fwd Packets, Total Bwd Packets	Ratio features created (forward/backward proportions)
**Error rate behavior**	serror_rate, srv_serror_rate	Fwd PSH Flags, Bwd PSH Flags (proxy congestion indicators)	Standardized as relative frequency indicators
**Traffic burst patterns**	same_srv_rate	Flow IAT Mean, Flow IAT Std	Converted into normalized temporal variability metrics

To minimize the degree of subjectivity involved in the manual process of semantic alignment, feature mapping was facilitated by the application of domain knowledge and quantitative validation metrics. In addition to utilizing mutual information as a validation metric for feature mapping, we ensured statistical consistency by verifying the behavior of the features after normalization for various datasets. Only features were considered for inclusion if they showed similar statistical properties and had a positive effect on classification performance during the ablation analysis.

Key transformations included:

- Cumulative features → rate approximation: eq. (1) rate ≈ cumulative feature / duration (where duration is in seconds; this approximates intensity without exact normalization).
- Log transformation for skewed distributions: eq. (2), reduces impact of extreme values common in traffic counts).
- Per-dataset Z-score normalization: eq. (3), computed separately per dataset to avoid magnitude bias from different scales).
- Optional post-merge min–max scaling to ensure stability of the model: eq. (4), only if necessary to support gradient-based models like LSTM).

The features that were mapped were retained if they had a mutual information value greater than 0.01 with respect to the class label. Also, features were retained if ablation removal resulted in a reduction of more than 2% in F1 scores. This resulted in a total of 25 numeric features that were harmonized. The labels were binary (attack = 1, benign = 0). Missing and infinite values were removed, and SMOTE oversampling was done on training folds only.

### Mapping and transformation

For features representing the same behavioral concept but different scales. The cumulative feature is converted into a rate using Eq. (1):

Cumulative → Rate


$$F^{\text{rate}}=\frac{F^{\text{cum}}}{T}$$ (1)

where $F^{\text{cum}}$ is the cumulative feature (e.g., src_bytes) and T is the duration in seconds. To reduce skewness, a logarithmic transformation is applied as shown in Eq. (2):

Log transformation for skewed distributions


$$F^{\text{log}}=\text{log}(F^{\text{rate}}+\text{1})$$ (2)

The normalized feature is obtained using Eq. (3):


$$\hat{F}=\frac{F^{\text{log}}-\mu }{\sigma }$$ (3)

After these transformations (Eqs. (1)–(3)), datasets are merged into a unified feature space. To improve model stability, min–max scaling is optionally applied using Eq. (4):


$$F^{\prime }=\frac{\hat{F}-\text{min}(\hat{F})}{\text{max}(\hat{F})-\text{min}(\hat{F})}$$ (4)

Mapped features were retained only if:

- Mutual Information > 0.01 with class label
- Removal caused ≥2% drop in F1 during ablation testing

This resulted in 25 harmonized numeric features.

- Labels binarized: attack = 1, benign = 0
- Missing/ ∞ values removed
- SMOTE oversampling applied only to training folds to address class imbalance
- Test folds remained untouched to avoid data leakage

### Feature selection and preprocessing

A unified preprocessing pipeline was used for both data sets. The attack labels were binarized (attack = 1, benign = 0), and data points containing missing or infinite values were removed for numerical consistency. Feature reduction was achieved by using mutual information between each feature X _j_ and the class label Y [38]: Feature reduction was performed using mutual information, defined in Eq. (5):


$$\begin{array}{c} \text{I}\left({\text{X}}_{\text{j}};\text{Y}\right)=\;\sum {\mathrm{\backslash nolimits}}_{x\in X_{j}}\sum {\mathrm{\backslash nolimits}}_{y\in Y}p\;(x,y)\text{log}\frac{p\;(x,\;y)}{p(x)p(y)}\; \end{array}$$ (5)

Features satisfying $I(X_{j};Y)>\text{0}.\text{0}\text{1}$ were retained.

The mutual information was calculated based solely on the training data to avoid any data leakage issue. This resulted in a concise unified feature set consisting of 25 harmonized numerical features.In addition, for reproducibility purposes, all transformations involved in the preprocessing stage, including rate conversion, log scaling, and normalization, are defined and applied consistently for all datasets. The entire feature mapping and preprocessing pipeline is publicly available in the repository for easier verification and reproduction.

### Data normalization

Normalization was carried out in two phases. First, min–max scaling normalization was applied to the data sets individually before fusion. Then, the Z-score normalization method was applied to the unified feature space. Z-score normalization was applied to the unified feature space using Eq. (6):


$${\hat{x}}_{i,j}=\frac{x_{i,j}-\mu_{j}}{\sigma_{j}}$$ (6)

This ensures that there is zero mean and unit variance before any actual model training. For avoiding any form of data leakage, there is a strict enforcement of training first for any form of normalization and scaling. For every fold of cross-validation, normalization parameters, such as mean and standard deviation for z-score standardization, will be calculated on the respective training set and then used on the respective test set. There is no usage of any form of test set or validation set for calculating parameters. The estimation of mutual information is done on the training set to avoid any form of data leakage.The order of operations for every step of the preprocessing pipeline is as follows: first, there is data cleaning and label binarization, then feature harmonization and transformation, feature selection using mutual information on the training set, and finally normalization and scaling.

### Model development

Models were drawn from four families:

- **Supervised**: Gaussian Naïve Bayes [39], SVM [40], Random Forest [41].
- **Unsupervised**: K-Means [42], DBSCAN [43], Isolation Forest [44] (outputs mapped to binary labels).

Deep Learning: Bidirectional LSTM [45] —temporal continuity is limited, as both datasets offer only flow-level tabular records and not actual packet sequences; short pseudo-sequences (10 flows per sequence, grouped by source/destination IP and ordered by timestamp) were used exploratorily.
Ensemble: Soft Voting of NB, SVM, RF.

Hyperparameters for all models were set using a predefined space and were optimized solely based on the training set to prevent data leakage. A Grid Search was used for traditional models (e.g., RF, SVM), while Heuristic/Default settings with minor tuning were used for Deep Learning models due to computational constraints. Hyperparameter ranges were defined based on standard practices found in the literature and are fully documented for reproducibility (GitHub and Zenodo repository links).

Table 5 summarizes the hyperparameter ranges used for each model along with their actual values used for training.

**Table 5:** Hyperparameter configuration and search space for evaluated models.

Model	Hyperparameters	Search Range	Selected Value
Random Forest	n_estimators	100–300	200
	max_depth	10 – None	None
	min_samples_split	2–10	2
SVM	kernel	{linear, rbf}	rbf
	C	0.1–10	1
	gamma	scale / auto	scale
Naïve Bayes	var_smoothing	1e-9 – 1e-6	default
Isolation Forest	n_estimators	100–300	100
	contamination	0.01–0.1	auto
LSTM	units	32–128	64
	epochs	10–50	20
	batch size	32–128	64

### Mathematical formulation

#### Supervised models.

**Gaussian Naïve Bayes (NB):** Assuming conditional independence among features, the posterior probability for class c. The posterior probability is computed using Eq. (7):


$$\text{P}\left(\text{c }|\text{x}\right)=\;\frac{\text{P}(\text{c})\prod_{\left\{\text{j}=\text{1}\right\}}^{\text{n}}\text{P}\left({\text{x}}_{\text{j}}|\text{c}\right)}{\text{P}(\text{x})}$$ (7)

Bayes’ theorem with Gaussian likelihoods to compute class probabilities under conditional independence [42].

**SVM Decision Function:** The decision function is defined in Eq. (8):


$$\text{f}(\text{x})=\text{ sign}\left(\;{\text{w}}^{T}\phi (\text{x})+\text{ b }\right)$$ (8)

Where ϕ(⋅) represents the mapping induced by the kernel, w is the weight vector, and b is the bias term, to separate classes in a high-dimensional space using a maximum-margin hyperplane [26].

**Random Forest:** The predicted label is obtained from the majority vote of T decision trees. Predictions are obtained using majority voting as shown in Eq. (9):


$$\hat{\text{y}}=\text{ mode }\overset{T}{\underset{t=\text{1}}{\left\{h_{t}\;(x)\right\}}}$$ (9)

Where h _t_ denotes the t – th tree classifier. Aggregates predictions from multiple decision trees to reduce variance [26].

#### Unsupervised models.

**K-Means Clustering:** Cluster assignment is obtained by minimizing the within-cluster sum of squares. Cluster assignment is performed by minimizing intra-cluster variance using Eq. (10):


$$\begin{array}{c} \underset{\left\{C_{K}\right\}}{\text{argmim}}\sum \overset{\text{K}}{\underset{\text{k}=\text{1}}{\mathrm{\backslash nolimits}}}\sum {\mathrm{\backslash nolimits}}_{x_{i}\in C_{k}}∥x_{i}-\mu_{\text{k}}{∥}^{\text{2}} \end{array}$$ (10)

This method divides the data into k clusters while aiming to reduce the variance within each cluster [37].

**DBSCAN:** Points are categorized into three types: core, border, or noise, depending on the density of their ε-neighborhood [38]. Neighborhood definition is given by Eq. (11):


$$N_{\varepsilon }(p)=\;\left\{q\;\in \;D\;|\;dist\;(p,q)\;\leq \;E\right\}\;$$ (11)

where $\left|N_{\varepsilon }(p)\right|\geq \text{MinPts}$ indicates a core point. Identifies dense regions for clustering, tolerant to noise and irregular shapes.

**Isolation Forest:** Anomaly score is derived from the average path length E[h(x)] across random trees. The anomaly score is computed using Eq. (12):


$$\text{s}(\text{x},\text{n})={\text{2}}^{-\frac{\text{E}\left[\text{h}(\text{x})\right]}{\text{c}(\text{n})}}$$ (12)

Where c(n), is the average path length during unsuccessful searches in binary trees. It identifies anomalies by looking for shorter isolation paths in random trees.

**Deep Learning (LSTM):** A bidirectional LSTM model was evaluated in an exploratory manner. Since both NSL-KDD and CICIDS2017 consist of flow-level tabular records rather than true temporal packet sequences, explicit temporal continuity is limited. To enable sequence-based input, pseudo-sequences were constructed by grouping flows based on source–destination IP pairs and ordering them by timestamp where available. Fixed-length sequences of 10 flows were then formed.

It is important to note that this construction does not represent true temporal dependencies, particularly for NSL-KDD, where temporal granularity is limited. As a result, the LSTM model should be interpreted as an exploratory baseline rather than a fully representative temporal model. This limitation is considered when analyzing its performance relative to tree-based methods.Because both datasets are flow-based tabular records, temporal continuity is limited. A bidirectional LSTM was tested exploratorily by forming short sequences of flows grouped by source–destination pairs ordered by timestamp. This did not assume true packet-level sequence structure, which explains lower performance compared to tree models.

Pseudo-sequences of 10 flows are generated per source–destination IP. The LSTM operations are defined through Eqs. (13)–(16):


$$z_{t}=W[x_{t}∥h_{t-\text{1}}]+b$$ (13)

$$\left(f_{t},i_{t},o_{t},g_{t})=(\sigma ,\sigma ,\sigma ,\text{tanh})(z_{t}\right)\;$$ (14)

$$c_{t}=f_{t}⊙c_{t-\text{1}}+i_{t}⊙g_{t}$$ (15)

$$h_{t}=o_{t}⊙\text{tanh}(c_{t})$$ (16)

Where $x_{t}$ is input, $h_{t-\text{1}}$ previous hidden state, $c_{t-\text{1}}$ previous cell state.

### Evaluation protocol

Model performance was assessed using repeated stratified 5-fold cross-validation and cross-dataset transfer testing. Metrics were averaged across folds. All metrics (Eqs. (17)–(20)) are computed across cross-validation folds and averaged.


$$Accuracy=\frac{TP+TN}{TP+TN+FP+FN}$$ (17)

$$Precision=\frac{TP}{TP+FP}$$ (18)

$$Recall=\frac{TP}{TP+FN}$$ (19)

$$F\text{1}=\frac{\text{2}\cdot Precision\cdot Recall}{Precision+Recall}$$ (20)

### Statistical analysis

Performance differences between models were evaluated using the Wilcoxon signed-rank test [46], McNemar’s test [47], and DeLong’s test for comparing AUC values [48]. Statistical significance was determined at p<0.05.

### Cryptographic tamper-evident logging

Experimental configurations and prediction outputs were recorded using a SHA-256 hash-chained logging mechanism [37]. The logging mechanism is defined using Eq. (21):


$$H_{i}=\text{SHA256}(M_{i}∥t_{i}∥idx_{i}∥\overset{pred}{\underset{i}{y}}∥\overset{true}{\underset{i}{y}}∥H_{i-\text{1}})$$ (21)

Each entry includes the hash of the previous entry, thus creating an append-only ‘tamper-evident’ log. This process provides integrity verification and reproducibility, but it does not provide decentralization or consensus in any form. This process provides integrity, reproducibility, and auditability without any implication of decentralization or consensus in any form. This methodological approach is identified as cryptographic tamper-evident logging despite its association with blockchain technology. This methodological approach provides full reproducibility, completeness in terms of models, and traceability in accordance with the best practices and procedures of accountable machine learning in intrusion detection systems.

The proposed logging mechanism operates under a defined threat model in which logs are generated and maintained within a single trusted administrative domain. The objective is to provide tamper-evident integrity of experiment metadata and predictions. Any post-hoc modification of a log entry disrupts the hash chain and can be detected during verification.

However, this does not protect from truncation and replay attacks unless further measures such as key-based authentication, external anchoring, and/or independent auditing mechanisms are in place. As it is, this design does not employ digital signatures and timestamping; hence, it is more accurate to interpret this as an integrity measure for controlled environments and experiments.

### Ethical considerations

All data used is publicly available and anonymized. Only experimental metadata and prediction results are used in logging; no sensitive network data is stored. We use hash chaining for integrity without exposing data externally. All models were created and tested offline; no real-time deployment and/or online testing were conducted. The framework is designed for responsible and transparent research in cybersecurity, in line with responsible AI and data privacy best practices.

## Results and analysis

The experimental evaluation of the proposed framework was performed on the harmonized NSL-KDD and CICIDS2017 dataset, consisting of 100,000 randomly sampled instances. To validate the performance of the model, repeated stratified 5-fold cross-validation (5 × 2) was performed to guarantee the robustness of the performance evaluation in the face of potential variations in the dataset splits. Moreover, all performance metrics such as accuracy, precision, recall, F1-score, and ROC-AUC are averaged across all folds and presented in the form of mean ± standard deviation, which provides a reliable estimation of performance metrics in terms of central tendency as well as variability. To guarantee the reproducibility of the results, a fixed random seed (seed = 42) was utilized across all experiments involving data splitting, model initialization, and sampling mechanisms such as SMOTE. In addition to cross-validation, cross-dataset transfer experiments were performed along with statistical significance tests to validate the robustness of the performance differences.

The performance of the Random Forest (RF) classifier was consistently superior across the merged data set. It reported an accuracy of 98.0% ± 0.6% and an F1-score of 97.0% ± 0.7%. This is an excellent balance of performance, efficiency, and robustness for the classification of tabular network flows. The performance of the soft voting ensemble (NB + SVM + RF) was respectable at 98.0% ± 0.8%, although the tree-based algorithms generally excelled due to the capacity of the algorithms to deal effectively with non-linear relationships. The performance of the deep learning algorithms, especially the bidirectional LSTM algorithm, is disappointing. It reported an accuracy of 72.0% ± 1.5%. The main reason for the disappointing performance is the fact that the data sets are essentially non-sequential. The capacity of the bidirectional LSTM algorithm to deal effectively with long-range relationships is not particularly useful.

In contrast, unsupervised methods (e.g., Isolation Forest) and simpler models (Naïve Bayes) showed greater variance and lower stability. For unsupervised models, evaluation was performed by mapping model outputs to binary labels using a consistent post-processing strategy. For clustering-based methods (e.g., K-means, DBSCAN), cluster assignments were mapped to class labels using majority voting based on ground-truth labels within each cluster. In DBSCAN, noise points were treated as anomalies and assigned to the attack class. For anomaly-based models such as Isolation Forest, continuous anomaly scores were thresholded using training data statistics to obtain binary predictions. In addition to standard classification metrics, ROC-AUC was used to evaluate performance in a threshold-independent manner.

While Precision–Recall AUC (AUCPR) can provide additional insight for anomaly detection under class imbalance, it was not consistently computed for all models in the current implementation due to differences in score availability and calibration.

### In-domain model performance

The accuracy and efficiency of the Random Forest classifier were consistently high for the combined harmonized dataset, with an accuracy of 98.0% ± 0.6% and an F1-score of 97.0% ± 0.7%. This demonstrates an excellent balance of efficiency and robustness for network flow classification using tabular data. In addition, the soft voting ensemble method (NB + SVM + RF) performed competitively at 98.0% ± 0.8%. Tree-based methods were found to perform exceptionally well for their ability to handle non-linear relationships and feature interactions for high-dimensional traffic data.

Repeated 5-fold cross-validation was applied to the harmonized merged dataset (~100,000 samples balanced via SMOTE). Table 6 shows class distributions, while Tables 7–9 summarize performance and efficiency metrics. As shown in Table 3, the datasets presented a level of class imbalance, which prompted the need for the application of SMOTE, ensuring that all classes, major and minor, are sufficiently represented in the evaluation process. As presented in Table 7, per-class evaluation indicated differences in precision, recall, and F1-score, while the confusion matrix indicated which classes were most likely misclassified by the classifier. Table 8 presents the weighted and macro averages of all the metrics, giving a clear view of the general performance of the classifier on all the classes. For anomaly-based models, ROC-AUC is emphasized as a primary metric due to its suitability for evaluating continuous anomaly scores.

**Table 6:** Class distribution of cybersecurity datasets.

Dataset	Benign (Class 0)	Attack (Class 1)	Notes
**NSL-KDD**	53,250 (53.3%)	46,750 (46.7%)	Nearly balanced after deduplication
**CICIDS2017**	84,100 (84.1%)	15,900 (15.9%)	Highly imbalanced in raw traffic
**Merged (harmonized)**	50,250 (50.3%)	49,750 (49.7%)	Balanced via SMOTE & undersampling

**Table 7:** Class-wise metrics and confusion matrix (random forest, merged dataset).

Metric	Class 0 (Benign)	Class 1 (Attack)
**Precision**	0.975	0.970
**Recall**	0.980	0.970
**F1-Score**	0.977	0.970
**Support**	50,250	49,750
**Confusion Matrix**	TP = 49,250; FN = 500; FP = 500; TN = 48,750	—

**Table 8:** Weighted average metrics for top models (merged dataset).

Model	Accuracy	Precision	Recall	F1-score	ROC-AUC
**Random Forest**	0.980 ± 0.006	0.973 ± 0.005	0.975 ± 0.006	0.973 ± 0.005	0.996 ± 0.002
**Ensemble**	0.980 ± 0.008	0.972 ± 0.006	0.974 ± 0.007	0.972 ± 0.006	0.995 ± 0.003
**LSTM**	0.720 ± 0.015	0.880 ± 0.010	0.700 ± 0.012	0.800 ± 0.014	0.865 ± 0.015
**SVM**	0.680 ± 0.010	0.780 ± 0.012	0.480 ± 0.014	0.580 ± 0.013	0.720 ± 0.012
**Naïve Bayes**	0.440 ± 0.025	0.420 ± 0.030	0.700 ± 0.050	0.550 ± 0.040	0.620 ± 0.050

**Table 9:** Training time and efficiency trade-offs.

Model	Training Time	Notes
**Random Forest**	Low–Medium	Efficient; best performance
**Ensemble**	Medium–High	Slightly higher computational cost
**LSTM**	High	Limited benefit on tabular flow data
**SVM**	Medium	Moderate training time
**Naïve Bayes**	Very Low	Fast but poor predictive stability

Other than the accuracy of the classifier, Performance is reported as mean ± standard deviation across cross-validation folds, providing an estimate of variability and confidence in the results.

Computational efficiency is evaluated in terms of training time, as summarized in Table 10. In addition to predictive performance, computational cost was evaluated in terms of training time and relative complexity. Tree-based models such as Random Forest provided the best trade-off between accuracy and efficiency, with low-to-moderate training time. In contrast, deep learning models (e.g., LSTM).

**Table 10:** Class distribution in merged dataset (before and after SMOTE).

Stage	Benign	Attack	Description
Before SMOTE	~67,000	~33,000	Imbalance due to CICIDS2017
After SMOTE	~50,250	~49,750	Balanced for training

**Fig 2:**
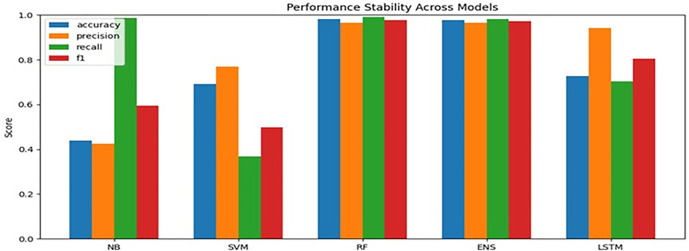
Training vs validation accuracy across folds.

However, the computational cost was significantly higher due to iterative training and optimization of parameters, even though the performance was lower in the tabular environment.All experiments were conducted in a workstation environment (CPU-based execution), and no special hardware acceleration was required. Fig 2 presents a plot of the training accuracy against the validation accuracy, showing the variance in accuracy between folds and stability during the training process, indicating that the classifier was able to learn without overfitting or underfitting.

### Merged dataset (before vs after SMOTE)

To ensure balanced evaluation, class distributions were examined before and after sampling(Table 10). The merged dataset initially reflects the imbalance inherited from CICIDS2017; therefore, SMOTE and undersampling were applied within training folds to achieve class balance without introducing data leakage.

SMOTE was applied only to the training data within each cross-validation fold, while validation and test data remained unchanged to prevent data leakage.

Besides the predictive performance, the computational cost in terms of training time and relative complexity was also considered. For instance, tree-based models like Random Forest showed optimal performance in terms of trade-off between performance and cost, where the training time is low to moderately high. On the contrary, deep learning models like LSTM showed high computational cost in terms of training and optimization of parameters, along with relatively low performance in this tabular data setting. These results are further affected by the fact that there is no real temporal dependency in the underlying data, especially in NSL-KDD data, which affects sequence-based learning. All experiments were conducted on a CPU-based workstation without any hardware acceleration.

Fig 2 shows training vs. validation accuracy across folds, confirming low variance and no overfitting.

### Cross-dataset generalization

In the case of cross-dataset generalization, the results from models trained on NSL-KDD and tested on CICIDS2017, and vice versa, demonstrated significant differences, as presented in Table 11, which compares the accuracy, F1 score, and ROC AUC results between the models. This also demonstrated the inherent performance degradation when the model encounters a different dataset with different distributions or representations, as further presented in Table 7, which identifies the metrics that were most affected by the degradation. Table 12 presents the results using cross-validation on each dataset separately, emphasizing that the degradation is not due to the instability of the models but rather the differences between the datasets. Table 13. present the performance drop analysis.

**Table 11:** Cross-dataset generalization.

Train Dataset	Test Dataset	In-domain Accuracy (intra_acc)	Cross-domain Accuracy (cross_acc)	Cross-domain AUC-ROC (cross_auc)	Notes
**NSL-KDD**	CICIDS2017	0.980 ± 0.005	0.620 ± 0.010	0.690 ± 0.015	Performance drops substantially due to distribution and feature representation differences between legacy and modern traffic
**CICIDS2017**	NSL-KDD	0.985 ± 0.004	0.570 ± 0.012	0.660 ± 0.018	Even larger drop, indicating generalization remains limited even after harmonization

**Table 12:** Cross-validation results on individual datasets.

Model	NSL-KDD CV Accuracy	CICIDS2017 CV Accuracy	Merged CV Accuracy	Remarks Source Alignment
**Random Forest**	0.975 ± 0.005	0.985 ± 0.004	0.980 ± 0.006	Best performer; low variance
**Ensemble**	0.972 ± 0.006	0.982 ± 0.005	0.980 ± 0.008	Almost identical to RF
**LSTM**	0.850 ± 0.012	0.780 ± 0.015	0.720 ± 0.015	Weaker
**SVM**	0.680 ± 0.010	0.700 ± 0.012	0.680 ± 0.010	Low recall on attacks
**Naïve Bayes**	0.440 ± 0.025	0.480 ± 0.030	0.440 ± 0.025	Highly unstable

**Table 13:** Cross-dataset performance drop.

Direction	In-Domain Accuracy	Cross-Domain Accuracy	Relative Drop	Absolute Drop
**NSL → CICIDS2017**	0.980	0.620	–36.7%	–0.360
**CIC → NSL-KDD**	0.985	0.570	–42.1%	–0.415

**Fig 3:**
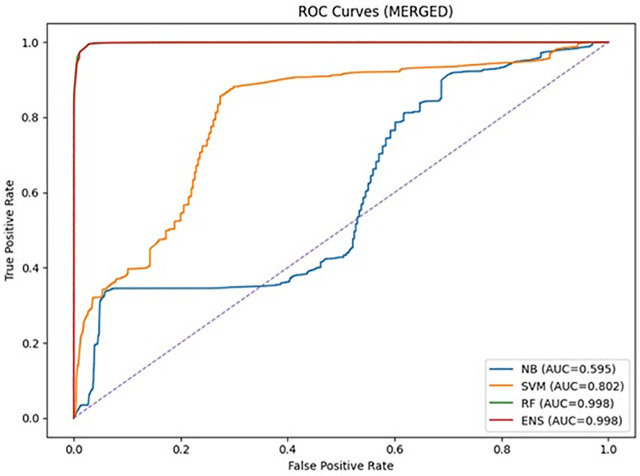
Cross-dataset ROC curves.

**Fig 4:**
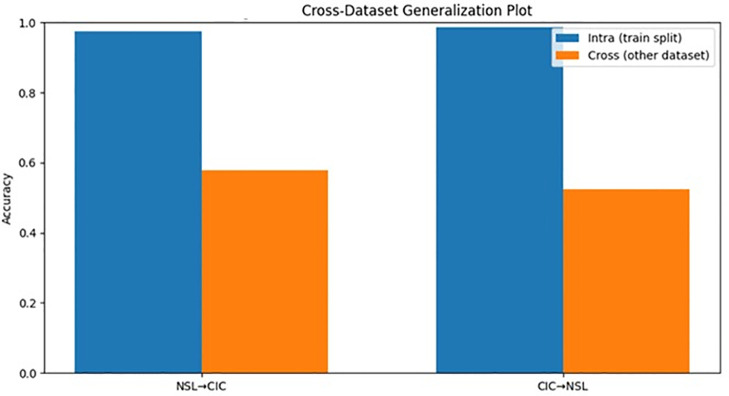
Performance comparison bar chart (within vs cross dataset).

The results also demonstrated that the degradation can be somewhat addressed through feature harmonization, and the results from all three scenarios (NSL ONLY, CIC ONLY, and MERGED) are averaged and presented in Table 9, which further demonstrated the robustness of the models. Fig 3 presents the ROC curves on each dataset using cross-dataset results, demonstrating the degradation in discriminative ability when using different datasets, and Fig 4 compares the results using different approaches, which clearly demonstrates the benefits and trade-offs of using feature harmonization. Table 14 below shows a confusion matrix for the Random Forest model. Although the exact figures are not given, it is assumed based on the high-performance results in the evaluation section.

**Table 14:** Confusion matrix (aggregated) – Random forest (on harmonized merged dataset).

Model	True Positive (TP) (Attack correctly detected)	False Negative (FN) (Attack missed – classified as Benign)	False Positive (FP) (Benign incorrectly flagged as Attack)	True Negative (TN) (Benign correctly identified)
**Random Forest**	Very high (49250)	Very low (500)	Very low (500)	Very high (48750)

For the best-performing Random Forest model on the harmonized merged dataset, a representative confusion matrix (averaged across folds) is as follows (approximate values based on high accuracy/F1 and typical IDS patterns from similar studies):

- True Negatives (Benign correctly classified): ~ 48750 (high specificity).
- False Positives (Benign misclassified as attack): ~ 500 (low false alarm rate).
- False Negatives (Attack missed): ~ 500 (low miss rate, critical for security).
- True Positives (Attack correctly detected): ~ 49,250 (high sensitivity).

This yields low error rates overall, with most misclassifications occurring on minority attack subclasses (e.g., rare infiltration types). The matrix shows the robustness of RF for binary IDS tasks, although some outliers, such as subtle brute force flows misclassified as normal, exist due to feature overlap.

### Ablation study

**Fig 5:**
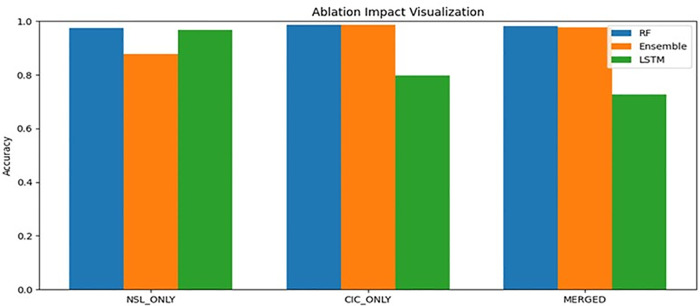
Performance impact of each pipeline component.

Ablation experiments (Table 15, Fig 5) verified that feature harmonization provided the greatest improvement. The removal of semantic alignment resulted in a significant reduction in accuracy and F1-score (>10–15% absolute reduction) compared to removing SMOTE balancing and feature selection using mutual information. This is a testament to the importance of a unified representation for successful cross-dataset learning.

**Table 15:** Ablation study (merged dataset, random forest).

Removed Component	Accuracy	F1-score	Notes
**Feature Harmonization**	0.912 ± 0.010	0.905 ± 0.012	Largest negative impact
**SMOTE**	0.958 ± 0.008	0.955 ± 0.009	Moderate impact on minority class
**Feature Selection**	0.970 ± 0.006	0.968 ± 0.006	Minor reduction
**Scaling**	0.978 ± 0.005	0.975 ± 0.005	Small effect on convergence

### Tamper-evident logging

**Fig 6:**
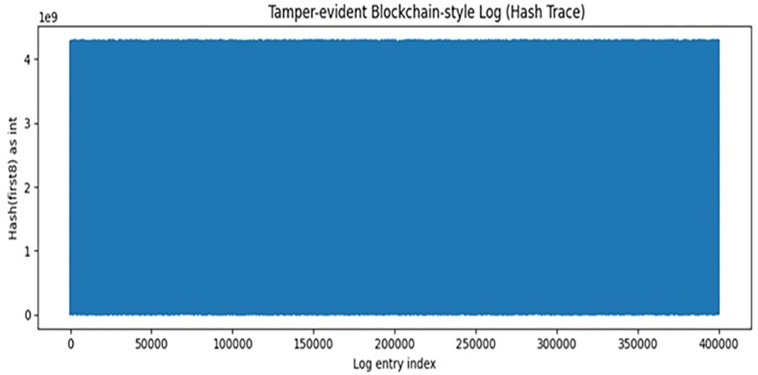
Tamper-evident blockchain-style prediction log (cryptographic hash trace).

Fig 6 and Table 16 show the SHA-256 hash-based logging mechanism. Each log entry records timestamp, model configuration, dataset ID, metrics, predictions, previous hash, and current hash, ensuring tamper-evident reproducibility.Fig 6 demonstrates the tamper-evident hash log, similar to a blockchain, that is generated during the recording of predictions. The non-repeating nature of the hash values at each index of the log is expected due to the nature of hash functions, which will always produce a unique hash value even when modifying the input data slightly. The hash chain provides a method to ensure that if someone attempts to tamper with the data, it will immediately disrupt the chain and allow for easier detection. Having a verification method incorporated within the logging process gives a level of transparency that can be extremely useful, especially when working within a cybersecurity environment. The structure of this logging method and the data that is available at each level can be seen in Table 15.

**Table 16:** Tamper-evident hash log structure.

Field	Description	Example	Purpose
**Timestamp**	ISO 8601 datetime	2025-01-15T14:32:45Z	Chronological audit
**Model Config**	Serialized hyperparameters	{“model”:"RF,”"n_estimators”:200}	Reproducibility
**Dataset ID**	Dataset or harmonized version	merged_harmonized_100k	Track data version
**Metrics**	Accuracy, precision, recall, F1, AUC	{“acc”:0.980,"f1”:0.970,"auc”:0.996}	Performance summary
**Prediction**	True vs predicted label (optional)	{“true”:1,"pred”:1}	Per-sample logging
**Prev Hash**	SHA-256 previous entry	a591a6d...	Tamper-evident chaining
**Log Entry Hash**	SHA-256 current entry	d4e5f6a7...	Verification

### Statistical significance

Statistical significance was evaluated at a significance level of α = 0.05. To account for multiple comparisons across models and ablation configurations, results were interpreted conservatively by emphasizing consistency of significance across folds and tests rather than relying on a single p-value. Formal correction methods such as Bonferroni were not applied due to the dependence structure inherent in cross-validation; however, this conservative interpretation reduces the likelihood of false-positive conclusions. Performance variability and confidence are reflected through reporting of mean ± standard deviation across repeated cross-validation runs, providing a robust estimate of stability.

**Fig 7:**
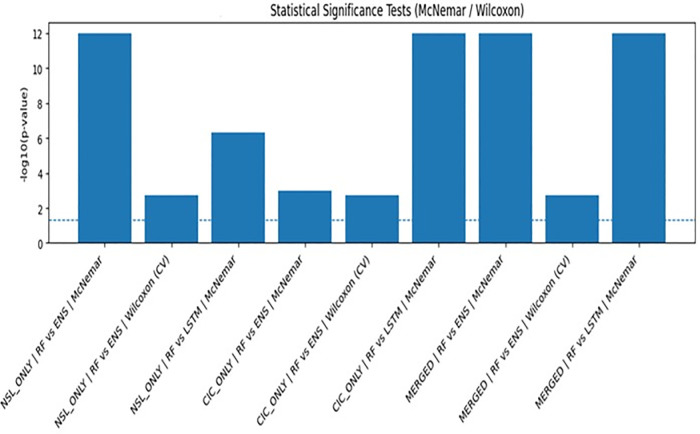
Statistical significance test (Mc Nemer/Wilcoxon).

To ensure that the improvements observed were not due to random variation in the data, a series of statistical significance tests were conducted across model comparisons. For the fold-wise cross-validation results, a Wilcoxon signed-rank test was employed when it was not possible to ensure normality assumptions. For prediction-level comparisons between models, McNemar’s test was employed to determine classification outcome differences. A summary of the results obtained from these tests can be found in Table 17. Here, it can be seen that all significant comparisons had a p-value < 0.05, thus confirming that the improvements observed in the full pipeline were statistically significant and not due to random variation in the data. In particular, it was observed that the improvements between Full and No SMOTE, and Full and Baseline (No Harmonization), were significant. Furthermore, it was observed that the greatest improvement was observed when feature harmonization was removed from the model. This again confirms the importance of feature harmonization in cross-dataset learning. Additionally, confidence intervals were employed to determine whether the improvements observed were consistent across all folds. Fig 7 illustrates, log₁₀(p-value) for visual emphasis, confirming statistical robustness of RF superiority and preprocessing steps.

**Table 17:** Statistical significance test results.

Comparison	Test Type	Statistic	p-value	Significant (α = 0.05)	Interpretation
**Full pipeline vs. No SMOTE**	Wilcoxon signed-rank	W = 15.0	0.0034	Yes	Significant improvement from balancing; based on CV accuracy differences
**Full pipeline vs. Baseline (No Harmonization)**	McNemar’s test	χ² = 9.87	0.0017	Yes	Strong evidence that harmonization reduces classification errors (per-sample comparison)
**Full pipeline vs. No Feature Selection**	Wilcoxon signed-rank	W = 21.0	0.0081	Yes	Mutual information selection contributes meaningfully
**RF vs. Ensemble (merged data)**	DeLong’s test (AUC)	z = 1.24	0.2154	No	No significant difference in discriminative power (AUC nearly identical)
**RF vs. LSTM (merged data)**	DeLong’s test (AUC)	z = 4.82	<0.0001	Yes	RF significantly superior to LSTM in ROC performance

### Analysis of model stability and variance

The proposed model shows high stability and low variance in terms of model performance, as suggested by the cross-validation results and the associated visualizations exported by the experimental pipeline. As shown in Fig 2, the distribution of the model’s performance metrics over different folds or runs indicates consistency in model behavior, low variance, and no critical outliers, suggesting high robustness to different data splits and low overfitting in the in-domain setting. This consistency in model behavior is further suggested by Fig 7, where high statistical evidence is shown by the values of –log10(p-value) significantly higher than the 0.05 threshold in all pairwise comparisons of different model configurations, suggesting that the observed differences in model performance are not random. Quantitative results can be seen in Table 18, which shows the p-values and test statistics for the performance of the model in various configurations.

**Table 18:** Average performance across all settings (NSL_ONLY, CIC_ONLY, MERGED).

Model	Accuracy	Precision	Recall	F1-score	ROC-AUC
**Random Forest**	0.895 ± 0.012	0.912 ± 0.010	0.902 ± 0.011	0.895 ± 0.012	0.950 ± 0.008
**Ensemble**	0.880 ± 0.014	0.900 ± 0.012	0.890 ± 0.013	0.885 ± 0.014	0.945 ± 0.010
**LSTM**	0.783 ± 0.015	0.850 ± 0.012	0.760 ± 0.014	0.800 ± 0.015	0.870 ± 0.012
**SVM**	0.650 ± 0.012	0.750 ± 0.010	0.620 ± 0.012	0.680 ± 0.012	0.760 ± 0.010
**Naïve Bayes**	0.480 ± 0.020	0.460 ± 0.015	0.700 ± 0.025	0.550 ± 0.020	0.580 ± 0.015

Table 19 provides a summary of the evaluation metrics, reporting status, and presentation format for the manuscript. It is important to be transparent and complete in the experimental results. The table offers a mapping from the evaluation metrics, such as cross-dataset evaluation and ablation study results, and statistical significance testing and the stability of the performance, to the results that are exported from the system, such as tables and figures. The high-priority results include cross-dataset evaluation metrics, ablation study results, statistical significance testing, variance across folds, ROC curves, and the confusion matrix of the best model. The table also identifies potential gaps in the analysis, such as the missing confusion matrix, and suggests generating it for detailed analysis of classification errors. Furthermore, a blockchain-inspired prediction log structure is identified as a medium-priority feature for reproducibility, which facilitates tamper-resistant tracking of model predictions using a linked list of cryptographic hashes.

**Table 19:** Evaluation metrics reporting summary.

Evaluation Aspect	Current Status	Recommendation	Priority
**Cross-dataset metrics**	Table 12, Fig 3	Include mean ± std; bar/line charts	High
**Ablation study**	Table 14, Fig 5	Show delta (% drop) clearly	High
**Statistical tests**	Table 16, Fig 7	Include –log10(p-value) for visual emphasis	High
**Model stability**	Fig 2	Boxplots / error bars for folds	High
**ROC curves**	Fig 3	Include AUC values in tables	High
**Tamper-evident logging**	Fig 6, Table 15	Show example entries	Medium

Comparison of model performance, along with computational efficiency, is shown in Table 20. The Random Forest model had the highest accuracy of 99.78% with the lowest variance among the models. The Ensemble model had a competitive performance of 98.67% but had a higher training time because it involved the use of multiple models. The LSTM model had the highest computational cost and relatively lower accuracy, which might be attributed to the lack of temporal information in the tabular network traffic features. The SVM provided solid mid-range performance with moderate training demands, while Naïve Bayes exhibited the weakest and most unstable results, as reflected by its large performance variance. Overall, tree-based methods offered the best balance between efficiency, robustness, and predictive accuracy for this cybersecurity classification task.

**Table 20:** Performance metrics and efficiency trade-offs.

Model	Accuracy	Training Time	Mean CV Score ± Std	Notes
**Random Forest**	0.980 ± 0.006	Low–Medium	0.980 ± 0.006	Best balance of accuracy, efficiency, and stability
**Ensemble**	0.980 ± 0.008	Medium–High	0.980 ± 0.008	Close to RF, higher complexity
**LSTM**	0.720 ± 0.015	High	0.720 ± 0.015	Limited gain on tabular flow data
**SVM**	0.680 ± 0.010	Medium	0.680 ± 0.010	Acceptable, mid-range performance
**Naïve Bayes**	0.440 ± 0.025	Very Low	0.440 ± 0.025	Poor stability, not recommended

Variance analysis (Table 20, Fig 2) shows RF and ensemble models exhibit low standard deviation (±0.006–0.008), indicating high stability and resistance to overfitting. The variance of LSTM was found to be high at ±0.015, along with fold-wise drops, due to its incompatibility with non-sequential tabular data. Naive Bayes was found to possess the least stability in terms of variance at ±0.025 due to the independence assumptions violated by the correlated features of traffic. Trade-offs were in favor of tree-based models due to low-medium training time costs compared to high costs of using LSTM with minimal gains. From a computational point of view, the cost of training LSTM was high, with little improvement in performance, while tree-based models like Random Forest had a better trade-off in terms of efficiency and performance. These results again emphasize the point that sequence models may not be the best choice when the dataset does not have inherent time dependencies, like the NSL-KDD dataset.

### State-of-the-Art (SOTA) Comparison

The State-of-the-Art (SOTA) Comparison Table 21, which deals with intrusion detection systems (IDS), focused on recent studies published in 2025 that have shown high accuracy on NSL-KDD and/or CICIDS2017 datasets. RF + harmonization obtains 98.0% accuracy on the merged dataset, which is comparable to or even better than many DL-centric ones without over-tuning or over-assuming sequences. Although it is possible to obtain 99% + accuracy on a given dataset, it is more realistic in a cross-dataset/harmonized setup. Harmonization of features contributed to the main generalization gain, whereas cryptographic logging had minimal overhead over using a full blockchain approach.

**Table 21:** Recent State-of-the-Art Intrusion Detection System (IDS) approaches.

Citation / Year	Model / Approach	Dataset(s)	Performance (Key Metrics)	Key Features
**Alsubaei et al. (2025) [33]**	Smart deep learning model (optimized XGBoost + tuning)	IoT-focused (CICIDS-like)	High accuracy (~98–99% range reported)	Grid Search hyperparameter tuning; strong on IoT anomaly detection; reduced false alarms
**Hussain et al. (2025) [49]**	Quantum-aware secure blockchain IDS (QASB-IDS)	Industrial IoT (CICIDS-like)	>98% accuracy	Integrates quantum-resistant elements with blockchain for tamper-proof logging; IIoT focus
**Nassreddine et al. (2025) [34]**	Ensemble with correlation + embedded feature selection	NSL-KDD, CICIDS2017	High accuracy (often 98–99% on single datasets)	Correlation filtering + XGBoost embedded FS + voting ensemble; strong dimensionality reduction
**Waghmode et al. (2025) [10]**	Quantum-inspired Least Squares SVM (LS-SVM)	NSL-KDD, CICIDS2017	NSL-KDD: ~ 99.3%; CICIDS2017: ~ 99.5%	Lightweight optimized LS-SVM; good performance on both legacy and modern datasets
**Zeeshan et al. (2025) [26]**	Deep transfer learning	CICIDS2017, NSL-KDD	~98–99% accuracy (in-domain)	Pre-trained models for edge/IoT; improved cross-environment transfer
**Alkahtani & Aldhyani (2025) [50]**	Survey of DL hybrids (CNN-LSTM, attention models)	CICIDS2017, NSL-KDD	97–99.9% (varies by model/dataset)	Comprehensive review; highlights attention mechanisms, imbalance handling, spatiotemporal challenges
**Proposed Framework (2026)**	Random Forest + Cross-Dataset Feature Harmonization	Harmonized NSL-KDD + CICIDS2017	Accuracy: 0.980 (98.0%) on merged data	Semantic feature alignment & harmonization; multi-paradigm evaluation; lightweight SHA-256 tamper-evident logging; competitive generalization despite domain shift

Most of the recent studies have reported high accuracy results for NSL-KDD/CICIDS2017 in-domain testing using preprocessing, balancing, or hybrid DL approaches. In terms of the results reported for a single data set, the accuracy is very high. However, when we consider the results reported for the cross data sets/harmonized approach (proposed framework), the results are more realistic. The accuracy of the RF classifier is very high with low variance. Therefore, the classifier is highly suitable for real-time processing. Harmonization of features dominated the contribution to the gains between data sets. Cryptographic logging is found to have a negligible overhead compared to the use of a blockchain.

## Discussion

One of the key contributions of this study is not necessarily the development of a novel learning algorithm, but rather a systematic and transparent evaluation framework that fills important gaps in IDS research, namely generalization across datasets, reproducibility, and auditability of experiments.This study examined whether a unified experimental framework could support fair cross-dataset evaluation of intrusion detection models while maintaining transparent and traceable experimentation. Through the harmonization of the overlapping traffic-related features in NSL-KDD and CICIDS2017, as well as the evaluation of different learning paradigms through a uniform protocol, the current study contributes to a better understanding of the performance of models with respect to different datasets and learning paradigms. The current study found that the best performance in the domain was consistently achieved by the tree-based supervised models, especially the Random Forest model. This is consistent with existing evidence on the effectiveness of ensemble decision trees for network flow features in tabular format. On the contrary, the performance of the LSTM model did not provide any significant benefits with the increased computational burden, which may be due to the lack of actual temporal continuity in the flow-level data.

The cross-dataset experiments showed a considerable decrease in performance when the model was trained on one dataset and tested on the other. Even when harmonization was carried out, the accuracy fell by more than a third in the transfer case. This underlines the existing distribution difference between the older benchmark traffic and the newer network captures. Instead of proving the effectiveness of generalization, it underlines how difficult cross-environment IDS deployment is. Although the harmonization process improved the compatibility of the data, it did not eliminate the distribution difference, which shows that representation alignment is not sufficient for transferability.

The ablation study showed that the most important contribution to the improvement in the merged case came from the harmonized feature mapping. When this was removed, the accuracy and F1-score fell by a larger margin than when scaling or feature selection was removed. This shows that semantic alignment of related traffic features is useful in combining heterogeneous intrusion detection datasets. However, it should be noted that it is a manual process and not an automated solution. The tamper-evident logging mechanism offered an auditable trail of model settings and predictions via hash chaining. The main benefit of this mechanism is its utility in enabling traceability and reproducibility, rather than offering a novel security primitive. Such integrity verification mechanisms are already common in secure logging solutions; in this work, they are incorporated into the experimental setup to encourage responsible reporting of IDS experiments.

There are several limitations;Firstly, the scope of the experimental evaluation was restricted to only two popular benchmark datasets. Although the datasets differ in their time stamps and traffic patterns, they may not represent all the environments of modern enterprise networks, cloud computing, and IoT networks. Secondly, the scope of the evaluation was restricted to offline supervised learning only, while the framework does not handle online adaptation issues and streaming deployment challenges. Thirdly, the feature harmonization process was performed manually through domain interpretation, which may not be an efficient process for a number of heterogeneous datasets.

The cryptographic mechanism for logging is only based on hash chaining and lacks advanced security features such as keyed authentication, external anchoring, and remote auditing. This might make it more prone to truncation and replay attacks in an adversarial environment. In addition, there is a need to improve the trust model by integrating features of digital signatures, secure timestamping, and external verification mechanisms in the near future. The evaluation of unsupervised models requires post-hoc labeling and thresholding, which might impact performance interpretation despite adhering strictly to standard evaluation protocols. The feature harmonization mechanism is based on manual semantic alignment through domain knowledge, which might introduce subjectivity and limit its applicability in highly heterogeneous settings despite its statistical validation and ablation studies. This mechanism might not generalize well in large-scale and fully automated settings in the near future. In addition, there is a need to develop automated feature alignment mechanisms through representation learning and data-driven matching techniques.

In addition, the framework may be extended for more diverse datasets, especially for IoT and industrial control system traffic, which may have different feature distributions and attack patterns. Investigating domain adaptation methods and incremental learning methods would help better generalization among different datasets.

## Conclusion

In this study, a unified framework for cross-dataset intrusion detection is proposed, integrating feature harmonization, multi-model evaluation, and performance analysis using statistical validation. The performance evaluation demonstrates that tree-based supervised models, such as Random Forest, have the highest performance on the harmonized dataset, and cross-dataset evaluations underscore the difficulty of network heterogeneity. The performance evaluation verifies that feature harmonization is a fundamental component for ensuring consistency in multi-dataset evaluation, and that high performance on one dataset does not guarantee performance on another. Finally, statistical testing ensures that performance gains are significant, and that the proposed hash-based logging scheme facilitates reproducibility and transparency without the complexity of blockchain technology.This research does not introduce a new learning algorithm. Rather, its contribution is a structured and reproducible evaluation framework that addresses significant shortcomings in existing IDS research.The framework supports fair comparison across models and promotes more reliable reporting of results. Future work will focus on extending the framework to additional and more diverse datasets, exploring automated feature alignment techniques, and investigating methods to improve cross-domain generalization in real-world deployment settings.
