# Correction: A cross-dataset harmonized intrusion detection framework with statistically validated multi-model learning

**DOI:** 10.1371/journal.pone.0349626

**Published:** 2026-05-20

**Authors:** The PLOS One Staff

There are errors in the Funding statement. The correct Funding statement is as follows: The authors extend their appreciation to the Deanship of Postgraduate Studies and Scientific Research at Majmaah University for funding this research work through project number (R-2026–133).

The publisher apologizes for the error.
